# Ebola Virus Disease Cluster in the United States — Dallas County, Texas, 2014

**Published:** 2014-11-21

**Authors:** Michelle S. Chevalier, Wendy Chung, Jessica Smith, Lauren M. Weil, Sonya M. Hughes, Sibeso N. Joyner, Emily Hall, Divya Srinath, Julia Ritch, Prea Thathiah, Heidi Threadgill, Diana Cervantes, David L. Lakey

**Affiliations:** 1 Epidemic Intelligence Service; 2 Division of Reproductive Health, National Center for Chronic Disease Prevention and Health Promotion, CDC; 3 Dallas County Health and Human Services; 4 Texas Department of State Health Services

Since March 10, 2014, Guinea, Liberia, and Sierra Leone have experienced the largest known Ebola virus disease (Ebola) epidemic with approximately 13,000 persons infected as of October 28, 2014 (1,2). Before September 25, 2014, only four patients with Ebola had been treated in the United States; all of these patients had been diagnosed in West Africa and medically evacuated to the United States for care.

On September 25, a man aged 45 years (patient 1), who had arrived in the United States from Liberia 5 days earlier, went to a Dallas County, Texas, emergency department with fever, initially 100.1°F (38.4°C) but increased to 102.9°F (39.4°C), abdominal pain, and headache (Figure). He was treated for possible sinusitis and discharged. On September 28, the man returned to the hospital by ambulance with persistent fever (101.4°F [38.6°C]), abdominal pain, and new onset diarrhea; he was placed in a private room under standard, droplet and contact precautions and was tested for Ebola. On September 30, real-time polymerase chain reaction (PCR) testing at the Texas Department of State Health Services and CDC confirmed that patient 1 was positive for Ebola virus, and this represented the first imported Ebola virus infection diagnosed in the United States. A CDC team arrived in Dallas later that night by invitation from the Texas Department of State Health Services to assist with its investigation. The objectives were to 1) identify potentially exposed contacts of the Ebola patient, 2) initiate monitoring of contacts, 3) review plans for triaging and testing suspected Ebola patients, and 4) review infection control practices.

Initial tracing of potentially exposed contacts (i.e., “contact tracing”) identified 48 close, unprotected contacts (i.e., had exposure to the patient, a potentially contaminated environment, or patient specimens without minimum recommended personal protective equipment [PPE]). Of the 48 contacts, 17 were persons within the community with exposure to the patient before he was admitted to the hospital and while he was symptomatic, 10 were persons who had been transported in the same ambulance that had transported the patient before it was completely cleaned and disinfected, and 21 were health care workers (HCWs) with potential exposures to body fluid without the protection of complete PPE. Beginning October 1, all 48 contacts underwent direct active monitoring (one in-person and one telephone follow-up per day to check for fever or symptoms of Ebola) for 21 days (the upper limit of the Ebola incubation period) from their last exposure date; six close community contacts were quarantined. Patient 1 died on October 8.

On October 11, a nurse (patient 2) previously involved in direct care of patient 1 developed fever (100.6°F [38.1°C]) and sore throat; she was confirmed to have Ebola by real-time PCR later that day. On October 14, a second nurse (patient 3) with similar exposure had a fever (100.5°F [38.1°C]) and rash and was confirmed to have Ebola by real-time PCR on October 15. Before her diagnosis, patient 3 had visited Ohio during October 10–13 (3). Contact tracing of patients 2 and 3 identified three household contacts of the two patients. Additional community contacts of patient 3 were identified from the Ohio visit and have been described (3).

Because patients 2 and 3 had used PPE during their care of patient 1 without reported exposures, all HCWs with any contact with any of the three Ebola patients, their laboratory specimens, or potentially contaminated environmental surfaces were interviewed beginning on the morning of October 12. All 147 HCW contacts of any of the patients, irrespective of PPE use, were actively monitored from October 12 until 21 days from their last exposure to an Ebola patient; those HCWs who had ever been in any of the three patients’ rooms were instructed not to use any long-distance and local public conveyances, and those who had ever been in patient 1’s room were additionally instructed not to attend mass gatherings (e.g., religious services). A subset of 20 HCWs volunteered to quarantine themselves.

In addition to contact tracing and monitoring, the Dallas Ebola investigation team 1) conducted technical consultations with five Dallas area hospitals to assist them in planning for providing care for confirmed Ebola patients; 2) established an emergency medical services transportation plan for known or suspected Ebola patients; 3) developed a plan for safely handling Ebola patient remains; 4) established capacity for PCR Ebola testing to be conducted at the Dallas County public health laboratory; 5) trained 160 HCWs on PPE use (e.g., proper selection and supervised donning and doffing) and infection control practices appropriate for caring for Ebola patients; and 6) helped establish a triage unit for evaluating contacts with symptoms compatible with Ebola. During this investigation, CDC developed new infection control guidance* and new guidance for assessing the risk of potential Ebola exposure.† By November 7, all 177 contacts of patients 1, 2, and 3 (some persons were contacts of more than one patient) completed 21 days of monitoring. In addition to patients 2 and 3, 12 persons who had contact with one or more of the Ebola patients were tested for Ebola after they developed fever or other symptoms potentially compatible with the disease during their monitoring period. Active monitoring aided in the prompt identification and evaluation of these contacts. None of those evaluated were found to have Ebola.

The Dallas Ebola cluster highlights many important issues that might be encountered by other jurisdictions in which an Ebola diagnosis is made locally, and for which jurisdictions should plan, including the need to 1) identify patients with Ebola at presentation to minimize potential exposures, 2) rapidly identify contacts of Ebola patients and evaluate their level of exposure risk, 3) monitor potentially large numbers of community and health care contacts, 4) assess infection control practices and conduct large-scale training sessions, 5) develop protocols to safely transport suspected Ebola patients to hospitals and safely evaluate these patients within a hospital, and 6) designate facilities to care for patients with confirmed Ebola.§

## Figures and Tables

**FIGURE:**
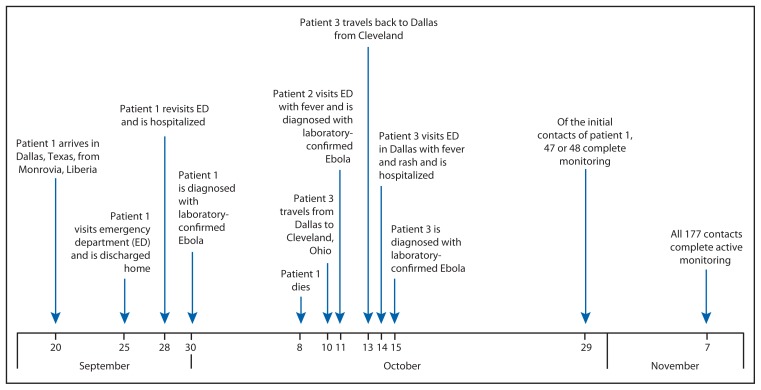
Timeline of events for Ebola patients 1, 2, and 3 — Dallas, Texas, September 20–November 7, 2014

## Footnotes

* Available at http://www.cdc.gov/vhf/ebola/hcp/infection-prevention-and-control-recommendations.html.

† Available at http://www.cdc.gov/vhf/ebola/exposure/monitoring-and-movement-of-persons-with-exposure.html.

§ Additional information available at http://www.cdc.gov/vhf/ebola.
